# 4'-O-β-D-glucosyl-5-O-methylvisamminol ameliorates imiquimod-induced psoriasis-like dermatitis and inhibits inflammatory cytokines production by suppressing the NF-κB and MAPK signaling pathways

**DOI:** 10.1590/1414-431X202010109

**Published:** 2020-10-30

**Authors:** Jing Fu, Zuping Zeng, Lu Zhang, Yan Wang, Ping Li

**Affiliations:** Beijing Hospital of Traditional Chinese Medicine, Capital Medical University, Beijing Institute of Traditional Chinese Medicine, Beijing Key Laboratory of Clinic and Basic Research with Traditional Chinese Medicine on Psoriasis, Beijing, China

**Keywords:** 4'-O-β-D-glucosyl-5-O-methylvisamminol, Lipopolysaccharide, Inflammatory cytokines, NF-κB, MAPK

## Abstract

Psoriasis is a chronic inflammatory skin disorder in humans, and the inflammatory reaction plays an important role in development and onset of psoriasis. 4'-O-β-D-glucosyl-5-O-methylvisamminol (4GMV) is one of the major active chromones isolated from *Saposhnikoviae divaricata* (Turcz.) Schischk, which has been reported to exhibit excellent anti-inflammatory activities. However, the possible therapeutic effect on psoriasis and underlying mechanism has not been reported. Thus, the aim of this study was to investigate the protective effect of 4GMV on the imiquimod (IMQ)-induced psoriasis-like lesions in BALB/c mice and the anti-inflammatory effect on the lipopolysaccharide (LPS)-induced inflammation in RAW264.7 macrophages. The results demonstrated that 4GMV decreased IMQ-induced keratinocyte proliferation and inflammatory cell infiltration. Moreover, 4GMV treatment significantly inhibited the production of NO, PEG 2, and cytokines such as interleukin (IL)-1β, IL-6, interferon (IFN)-γ, and IL-22 in LPS-stimulated RAW264.7 macrophages. 4GMV also suppressed the LPS-upregulated protein expressions of iNOS and COX-2 in a dose-dependent manner. Furthermore, qRT-PCR analysis showed that 4GMV down-regulated the mRNA level of IL-1β and IL-6 expression. Further studies by western blot indicated that 4GMV inhibited the activation of upstream mediator NF-κB by suppressing the expression of TLR4 and the phosphorylation of IκBα and p65. The phosphorylation of JNK, p38, and ERK were also markedly reversed by 4GMV in LPS-treated RAW264.7 macrophages. Taken together, these results demonstrated that 4GMV showed a protective effect in IMQ-induced psoriasis-like mice and inhibited inflammation through the NF-κB and MAPK signaling pathways, indicating that 4GMV might be a potential therapeutic drug for psoriasis.

## Introduction

Psoriasis, a chronic immune-mediated inflammatory skin disease, is characterized by thickened epidermis and scaly reddish plaques due to hyperproliferation and abnormal differentiation of keratinocytes (1). The pathogenesis of psoriasis is complicated and remains unclear. However, it has also been confirmed that macrophage infiltration and inflammatory mediators are related to the occurrence and development of psoriasis-induced skin inflammation (2).

Inflammation is a biological defense response of the immune system against harmful stimuli including damaged cells, irritants, and bacteria (3). Macrophages are the main effector cells of the immune system and play an important role in host defense against infectious microorganisms by producing inflammatory mediators, including cytokines (4). However, imbalanced inflammation may induce cellular and tissue damage in different diseases such as atherosclerosis, hypertension, diabetes, cancer, and neurodegenerative disorders (5–7). Lipopolysaccharide (LPS), an endotoxin component released by Gram-negative bacteria, induces a downstream signaling cascade through stimulation of the toll-like receptor 4 (TLR4) on the cellular surface of macrophages leading to the activation of nuclear factor kappa-light-chain-enhancer of activated B cell (NF-κB) (8). Once activated, NF-κB is translocated into the nucleus, where it binds to the promotor region of its target genes and induces the release of pro-inflammatory mediators such as tumor necrosis factor-α (TNF-α), interleukin (IL)-1β, IL-6, and nitric oxide (NO) (9). Furthermore, mitogen-activated protein kinase (MAPK) can be activated in LPS-induced macrophages and regulate inflammatory actions and immune responses (10). Therefore, the inhibition of NF-κB and MAPK pathways might be useful for the treatment of psoriasis.

**Figure 1:**
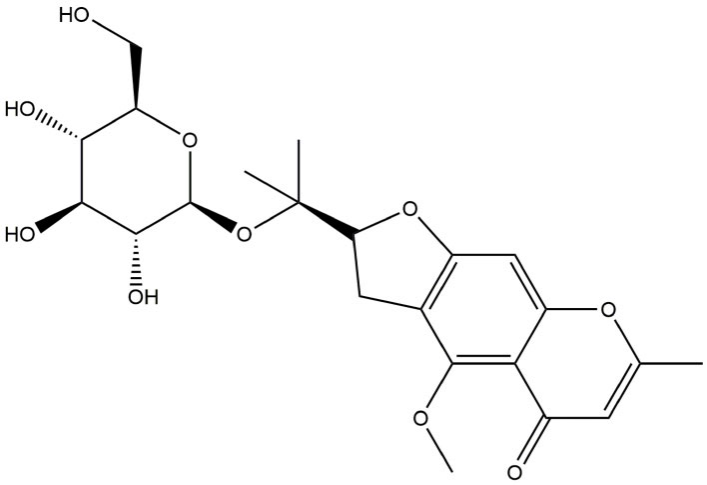
Chemical structure of 4'-O-β-D-glucosyl-5-O-methylvisamminol (4GMV).

Currently, topical treatment, systemic medications, and phototherapy are mainly used for the treatment of psoriasis (11). However, these methods also lead to numerous side effects. Therefore, there is an urgent need to find an agent with therapeutic effects and low toxicity for the treatment of psoriasis. Numerous products of natural origins with anti-inflammatory activities and minimal side effects are increasingly appreciated. *Saposhnikoviae divaricata* (Turcz.) Schischk has been used as traditional medicine in many countries, such as China (called Fang Feng), Japan (called Bou-hu), and Korea (called Bangpung) (12,13). Fang Feng possesses a wide range of biological and pharmacological activities, such as analgesic, anticonvulsant, anticancer, anti-inflammatory, anticoagulant activities, etc. (14). These pharmacological properties lay the foundation for the treatment of various diseases, including pyrexia, rheumatism, arthralgia, general aches, headaches, stroke, and allergic rhinitis (12). Abundant compounds have been isolated from it, such as chromones, conmarins, and polyacetylenes (15,16). 4'-O-β-D-glucosyl-5-O-methylvisamminol (4GMV) (Figure 1) is one of the major active chromone in its roots. It was reported that this compound exhibits obvious analgesic, anti-inflammatory, and antiplatelet aggregation effects (12). However, it is not currently known if 4GMV functions in psoriasis-like dermatitis induced by imiquimod (IMQ). Moreover, its anti-inflammatory mechanism has not been fully determined. Hence, in this study, we evaluated the anti-psoriasis effect of 4GMV as well as the roles of NF-κB and MAPK signaling pathways in its potential mechanisms.

## Material and Methods

### Drugs and chemicals

4GMV (Batch No. 111523‐201610, purity >96.10%) was obtained from Chinese Food and Drug Inspection Institute (China) and IMQ cream from Sichuan Mingxin Pharmaceutical Co. (China). Dulbecco's modified Eagle's medium (DMEM) and fetal bovine serum (FBS) were purchased from Corning (USA). LPS (from *E. coli*, isotype 055: B5) was obtained from Sigma Chemical Co. (USA) and PBS from Solarbio (China). Cell counting kit (CCK)-8 was purchased from Dojindo (Japan), nitric oxide assay kit from Applygen Co. (China), and prostaglandin E2 (PGE2) assay kit from Tianjin Createch Biotechnology Co. (China). Total RNA Extraction kit and One-step RT-PCR kit were obtained from Invitrogen (USA). Antibodies for TLR4, p-ERK, p-IκBα (nuclear factor of kappa light polypeptide gene enhancer in B-cells inhibitor, alpha), and p-p65 were purchased from Cell Signaling Technology (USA) and antibodies for β-actin, cyclo-oxigenase-2 (COX-2), inducible nitric oxide synthase (iNOS), TLR4, p38, p-p38, extracellular signal-regulated kinase (ERK), p-ERK, c-Jun N-terminal kinase (JNK), p-JNK, p-IκBα, and p-p65 were purchased from Abcam (UK). Methotrexate (MTX) was obtained from Shanghai Pharmaceutical Co. (China) and Vaseline from Lanlianfeitian Petrochemical Co. (China).

### Establishment of psoriasis animal model and administration of 4GMV

BALB/c mice (18-20 g) were purchased from Beijing Huafukang Biological Technology Co., Ltd. (China), and housed under specific pathogen-free conditions. All animal experiments were approved in accordance with the Animal Ethics Committee of the Beijing Institute of Traditional Chinese Medicine (No. 2019070201).

The mice were randomly divided into five groups: control, model, MTX (1 mg/kg), 4GMV low (6 mg/kg), and 4GMV high (12 mg/kg). According to our previous study (17), the mice received a daily topical dose of 62.5 mg of 5% IMQ to the shaved area (2×3 cm) on their backs in the model, MTX, and 4GMV low and high groups for 7 consecutive days to induce a psoriasis model. Mice in the control group were treated with Vaseline instead of IMQ.

### Severity index (PASI) assessment

The objective scoring system Psoriasis Area and Severity Index (PASI) was used to evaluate the severity of back skin inflammation, which consists of measurements for skin erythema, scaling, and thickness. Each parameter was scored independently on a scale from 0 to 4: 0, none; 1, slight; 2, moderate; 3, marked; and 4, very marked. The cumulative score denotes the severity of inflammation.

### Histological analysis

After 7 days, the mice were sacrificed by cervical dislocation under sodium pentobarbital (40 mg/kg, *ip*), and the skin lesions were collected, fixed in 10% formalin solution, and embedded in paraffin. The paraffin-embedded sections were stained with hematoxylin and eosin (H&E) for pathological observation by light microscopy. Epidermal thickness was measured by ImagePro Plus software (Leeds Precision Instruments, USA).

### Cell culture and treatment

RAW264.7 cells (Beijing Union Medical University, China) were maintained in high glucose DMEM containing 10% FBS and 1% antibiotics (100 U/mL penicillin and 100 μg/mL streptomycin) in a humidified incubator with 5% CO_2_ at 37°C. RAW264.7 cells were allowed to adhere in the logarithmic growth phase for 24 h before treatment. For all *in vitro* assays, 4GMV was dissolved in DMEM to a stock concentration of 0.5 mM and stored at 4°C.

### Cell viability assay

The effect of 4GMV on RAW264.7 cells was assessed using a CCK-8 assay. RAW264.7 cells were seeded in 96-well plates at a density of 1.0×10^4^ cells/well overnight. The cells were incubated (37°C) with fresh medium containing various concentrations of 4GMV (0, 25, 50, 100, 200, 500 μM) for 24 h. Then, 100 μL fresh medium and 10 μL CCK-8 were added to each well. The cells were further incubated at 37°C for 4 h. The absorbance of the samples was measured at 450 nm using a microplate reader (Thermo Scientific Multiskan GO, USA). All studies were performed in triplicate.

### NO production assay

The levels of NO were determined using Griess reagent (N-(1-naphthyl) ethylenediamine and sulfanilic acid), as previously described. Briefly, RAW264.7 cells were pretreated with different concentrations of 4GMV (0, 50, 100 µM) for 1 h before treatment with 1 μg/mL LPS. After 24 h, the culture supernatants were mixed with an equal volume of Griess reagent. The absorbance was measured at 540 nm using a microplate reader. The experiments were performed in triplicate.

### ELISA assays

Commercial ELISA kit was used for the measurement of PGE2 levels in the culture medium. RAW264.7 cells were pretreated with 4GMV (0, 50, 100 µM) for 1 h prior to LPS (1 μg/mL) treatment. The cell culture supernatants in each group were collected and stored -20°C prior to use. The concentration of PGE2 was detected at 450 nm using a microplate reader following the manufacturer's protocols. The experiments were performed in triplicate.

### Multiplex cytokine assays

The culture supernatants incubated with 4GMV were analyzed using a multiplex cytokine assay (Bio-Plex Human Cytokine 27-Plex Panel, Bio-Rad Laboratories, USA) containing the following analytes: IL-1β, IL-2, IL-3, IL-4, IL-5, IL-6, IL-9, IL-10, IL-12, IL-13, IL-17, IL-18, IL-22, IL-23, IL-27, interferon gamma (IFN-γ), TNF-α, and granulocyte-macrophage colony stimulating factor (GM-CSF) (18). The analysis was performed according to the instructions from the manufacturer.

### Quantitative RT-PCR analysis

Total RNA from RAW264.7 cells was extracted using TRIzol^®^ reagent as described before (19). To detect the effect of 4GMV on gene expression in LPS-stimulated cells, RAW264.7 cells were plated into 6-well plates (1.0×10^6^ cells/well) and pretreated with 4GMV for 1 h prior to LPS (1 μg/mL) treatment. After treatment for 24 h, RAW264.7 cells were collected and total RNA was isolated using TRIzol reagent solution according to the manufacturer's instructions. β-actin was amplified in parallel with the target genes and used as a normalization control for total messenger RNA (mRNA). The following PCR primer sequences (forward and reverse, respectively) were used: CGTTGACATCCGTAAAGACCTC and ACAGAGTACTTGCGCTCAGGAG for β-actin; GATAACAAGAAAGACAAAGCCAGAGTC and AGCATTGGAAATTGGGGTAGGAAG for IL-6; TGCCACCTTTTGACAGTGATGA and TGTGCTGCTGCGAGATTTGA for IL-1β. Data were analyzed by normalizing with GAPDH mRNA expression.

### Western blot analysis

After treatment with 4GMV, RAW264.7 cells were harvested and washed twice with ice-cold PBS. The cells were then lysed with ice-cold RIPA buffer for 30 min. The lysates were centrifuged at 12,000 *g* for 10 min at 4°C. Total protein concentration of the supernatant was determined by a BCA protein assay kit (Beijing Beyotime Institute of Biotechnology, China). Equal amounts of protein were separated on sodium dodecyl sulfate-polyacrylamide gels (SDS-PAGE) and then transferred to a polyvinylidene fluoride (PVDF) membrane. The membranes were blocked with 5% skim milk in Tris-buffered saline Tween-20 (TBST) buffer [25 mM Tris (pH 7.4), 150 mM NaCl, 0.1% Tween 20] for 1 h, and then incubated with appropriate primary antibodies overnight at 4°C. After three washes with TBST, the membranes were further incubated with corresponding secondary antibodies (20). An enhanced chemiluminescence (ECL) method was used for visualization of target proteins. The protein expression levels were measured by Quantity One analysis software (USA) using β-actin as an internal standard to normalize protein amounts. The experiments were repeated at least thrice.

### Statistical analysis

The experimental results were repeated in triplicate and data are reported as means±SD. Differences between mean values of normally distributed data were analyzed by one-way ANOVA and LSD test using the SPSS software (17.0; IBM, USA). P<0.05 were considered statistically significant.

## Results

### 4GMV activated IMQ-induced keratinocyte growth and inflammatory cell infiltration

**Figure 2:**
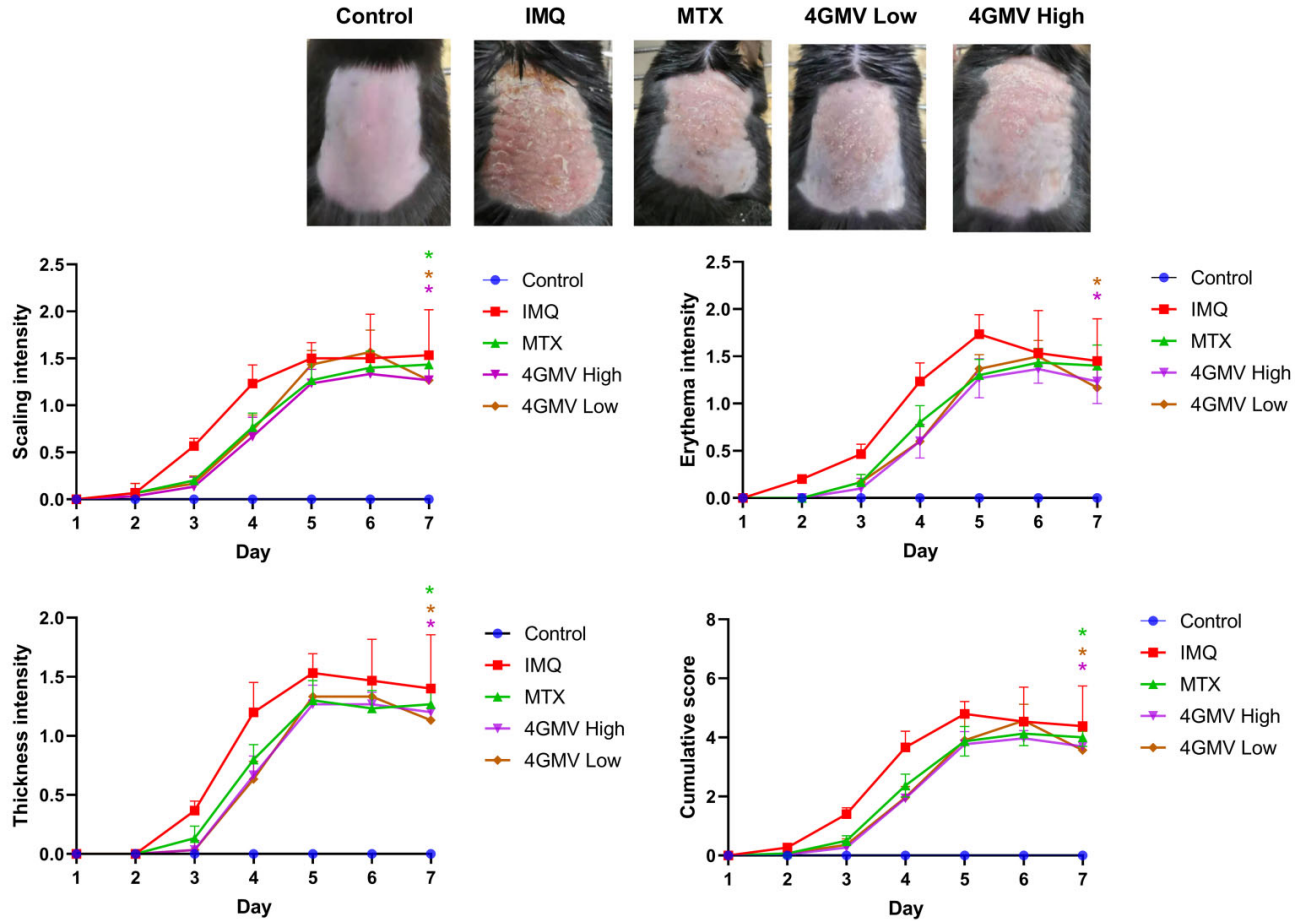
A mouse model of psoriasis was induced by topical application of imiquimod (IMQ). **Top**: Psoriasis-like skin lesions were observed after 7 days in the IMQ-treated group. Animals administered methotrexate (MTX) (1 mg/kg), 4GMV low (6 mg/kg), and 4GMV high (12 mg/kg) showed ameliorated symptoms. **Bottom**: Scaling, erythema, and thickness of the back skin was scored on a scale from 0 to 4. The cumulative Psoriasis Area and Severity Index (PASI) is also depicted. Data are reported as means±SD (n=6 per group). *P<0.05 compared with the IMQ model group (ANOVA).

Compared with the control group, the mouse skin in the model group exhibited the most severe lesions after 7 days. Animals treated with 4GMV and MTX showed shallow erythema, smoother skin, and sparser scales (Figure 2 top). Moreover, the control group exhibited no obvious changes in PASI scores. However, the psoriasis-like lesions gradually increased in the model group. Treatment with 4GMV (high and low doses) and MTX reduced the PASI scores compared to the model group. The thickening and cumulative score of 4GMV (high doses) groups roughly overlapped with that of the MTX group (Figure 2 bottom).

**Figure 3:**
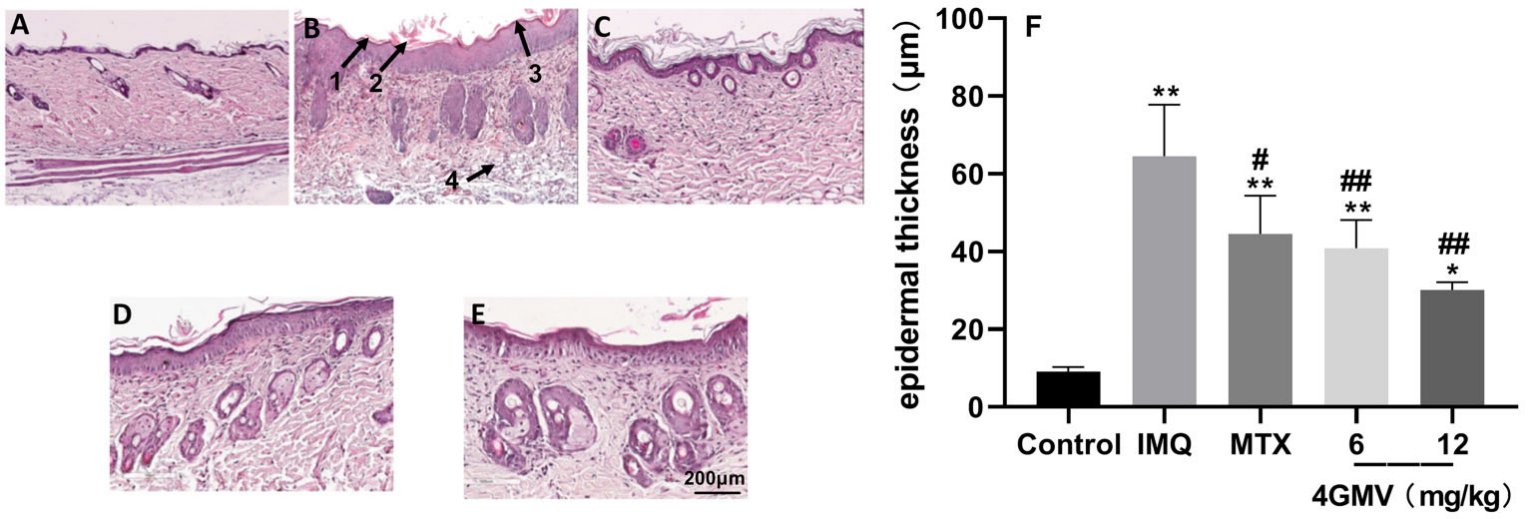
Histological evaluation of the back skin of imiquimod (IMQ)-induced psoriasis-like mice (staining with HE; magnification 200×, scale bar 200 µm). **A**, Control group; **B**, Model group: arrows 1-4 indicate epidermal hyperplasia, hyperkeratosis, parakeratosis, and inflammatory cell infiltration, respectively; **C**, methotrexate (MTX) group (1 mg/kg); **D**, 4GMV low group (6 mg/kg); **E**, 4GMV high group (12 mg/kg). **F**, Epidermal thickness of skin lesions in mice (n=6). Data are reported as means±SD. *P<0.05 and **P<0.01 compared with the control group; ^#^P<0.05 and ^##^P<0.01 compared with the IMQ group (ANOVA followed by LSD).

The mice treated with IMQ exhibited pathological psoriatic lesions, including epidermal hyperplasia, hyperkeratosis, parakeratosis, as well as inflammatory cell infiltration in the model group. The 4GMV and MTX groups had significantly reduced thickness of the epidermis layer, and attenuated IMQ-induced psoriasis conditions, including smoother epidermis, less epidermal thickening, and less parakeratosis (Figure 3A). In the model group, microscopy showed that the vertical epidermis thickness was about 7 times greater than in the normal control group. In addition, the epidermal thickness in MTX and 4GMV groups was significantly less (Figure 3B).

### Effect of 4GMV on viability of RAW264.7 cells

**Figure 4:**
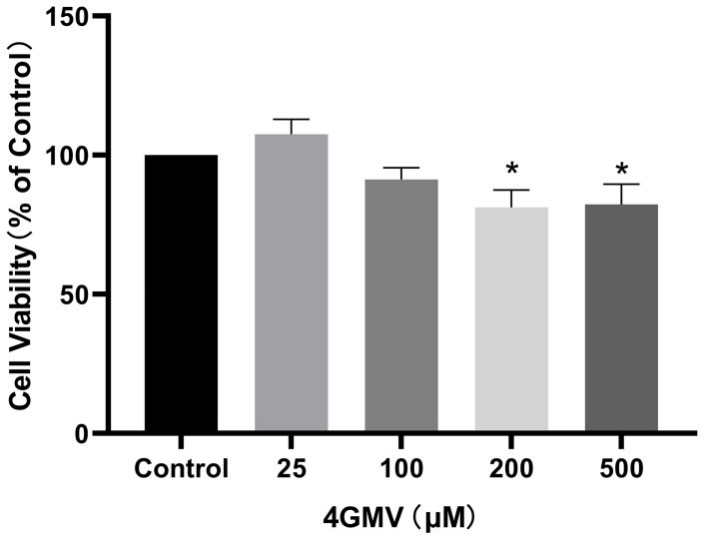
Effect of different concentrations of 4'-O-β-D-glucosyl-5-O-methylvisamminol (4GMV) (0, 25, 50, 100, 200, 500 μM) on cell viability in RAW 264.7 cells was detected by CCK-8 assay. Data are reported as means±SD from three independent experiments (n=3). *P<0.05 compared with the control group (ANOVA followed by LSD).

Compared with the vehicle controls, the results showed that 4GMV at concentrations from 25 to 100 μM had no cytotoxic effect on RAW264.7 cells (Figure 4). Therefore, concentrations of 50 and 100 μM 4GMV were selected for further experiments.

### Effect of 4GMV on LPS-induced inflammation

**Figure 5:**
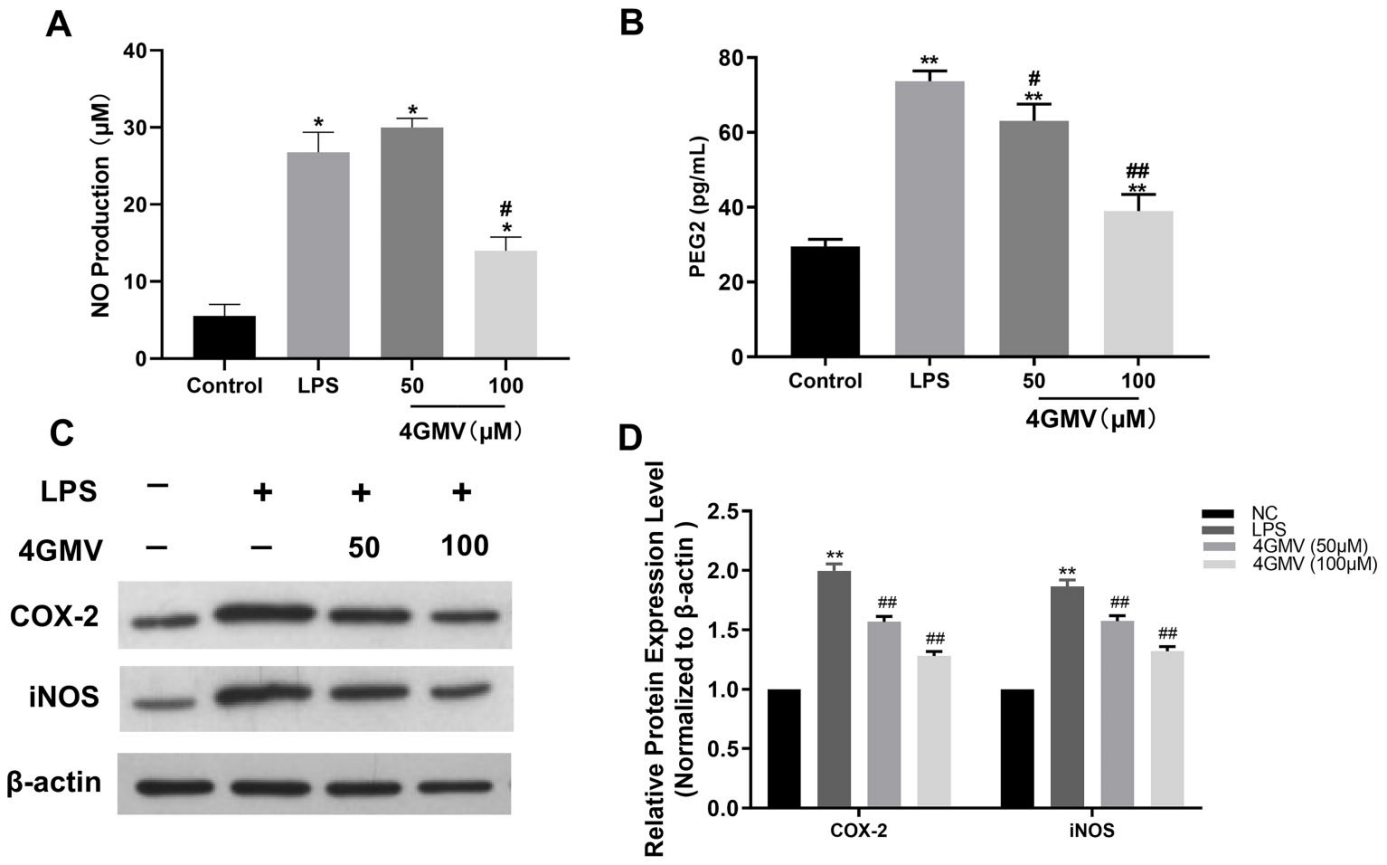
Effect of 4'-O-β-D-glucosyl-5-O-methylvisamminol (4GMV) on the expression of nitric oxide (NO), prostaglandin E2 (PGE2), cyclo-oxigenase-2 (COX2), and inducible nitric oxide synthase (iNOS) in RAW 264.7 cells. RAW 264.7 cells were pretreated with different concentrations of 4GMV (50 and 100 μM) for 1 h prior to treatment with 1 μg/mL lipopolysaccharide (LPS). **A**, NO production was measured by the Griess test. **B**, The production of PGE2 was detected in the culture supernatants. **C**, Protein expression of COX-2 and iNOS was examined by western blotting. **D**, Quantitation of band density. Data are reported as means±SD (n=3) from three independent experiments. *P<0.05 and **P<0.01 compared with the control group; ^#^P<0.05 and ^##^P<0.01 compared with the LPS group (ANOVA followed by LSD). NC: negative control (β-actin).

In order to investigate the anti-inflammatory property of 4GMV, we detected its effect on LPS-induced inflammatory responses by measuring the accumulated nitrite in the culture medium as estimated by Griess reaction. NO significantly increased in LPS-stimulated cells compared with the control group. However, pretreatment with 4GMV (100 μM) significantly decreased NO concentration compared to the LPS group (Figure 5A). 4GMV also significantly downregulated LPS-induced overexpression of PGE2 in RAW264.7 cells in a concentration-dependent manner (Figure 5B).

### Effect of 4GMV on LPS-induced COX-2 and iNOS in RAW264.7 cells

To clarify the molecular mechanism underlying the inhibition of LPS-induced NO and PGE2 production by 4GMV (50 and 100 μM), the expression levels of iNOS and COX-2 were evaluated, which are important upstream regulators of the expression NO and PGE2, respectively (21). As shown in Figure 5C and D, LPS challenge significantly increased the expression of iNOS and COX-2 proteins, while 4GMV pretreatment attenuated the overexpression of iNOS and COX-2 in a dose-dependent manner.

### Effect of 4GMV on LPS-induced generation of cytokines in RAW264.7 cells

**Figure 6:**
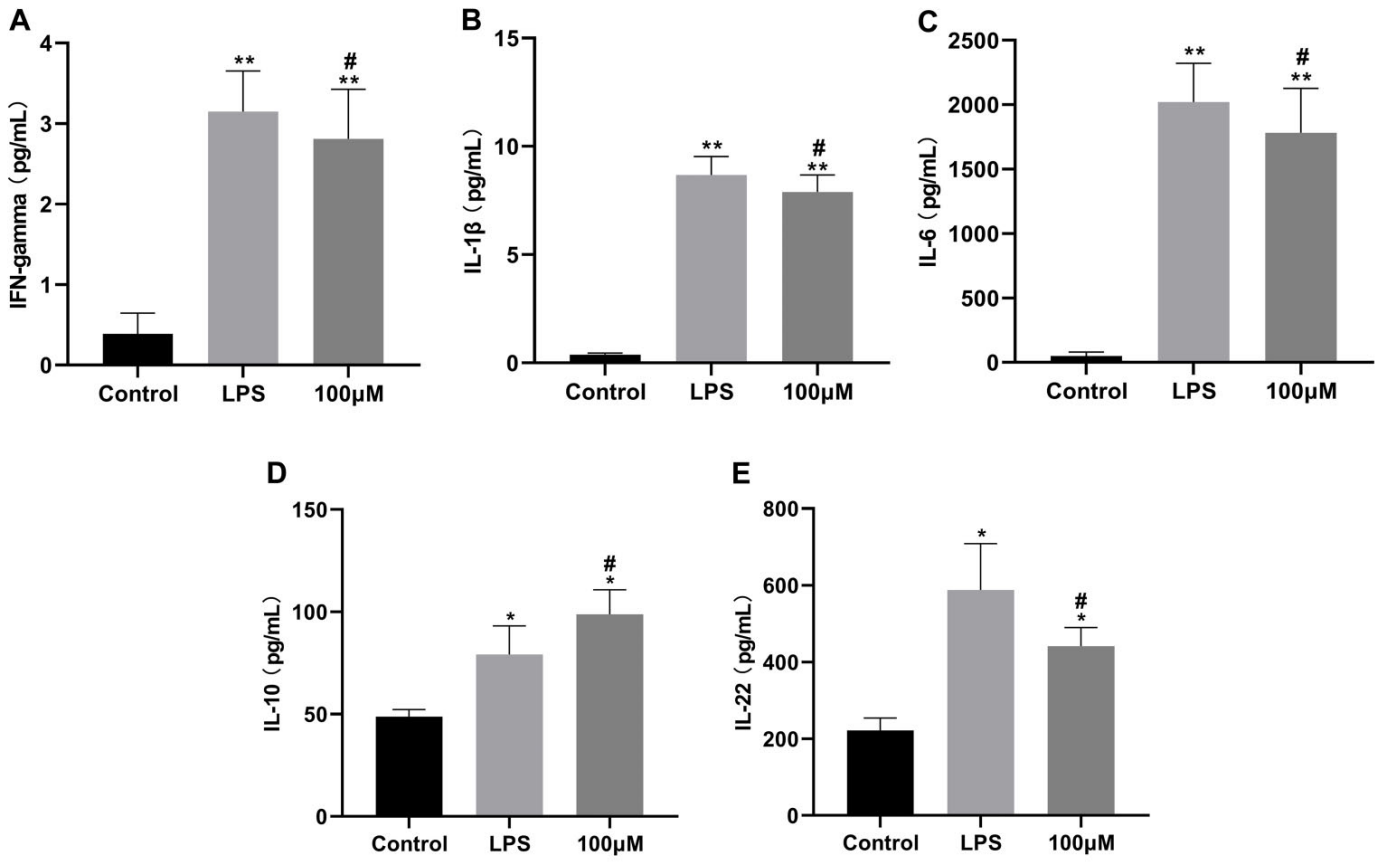
Effect of 4'-O-β-D-glucosyl-5-O-methylvisamminol (4GMV) on lipopolysaccharide (LPS)-induced production of cytokines in RAW 264.7 cells. RAW 264.7 cells were pretreated with 4GMV (100 μM) for 1 h prior to treatment with 1 μg/mL lipopolysaccharide (LPS). After 24 h, the culture supernatants were analyzed using a multiplex cytokine assay. **A**-**E**, Production of interferon (INF)-γ, interleukin (IL)-1β, IL-6, IL-10, and IL-22, respectively. Data are reported as means±SD (n=3) from three independent experiments. *P<0.05 and **P<0.01 compared with the control group; ^#^P<0.05 compared with the LPS group (ANOVA followed by LSD).

Compared with the LPS group, the levels of IL-1β, IL-6, IFN-γ, and IL-22 were significantly reduced after exposure to 4GMV (100 μM). However, the production of IL-10, an anti-inflammatory cytokine, was increased (Figure 6). Other cytokines did not show a significant inhibitory effect.

### Effect of 4GMV on LPS-induced IL-6 and IL-1&mac_bgr; mRNA expressions in RAW 264.7 Cells

**Figure 7:**
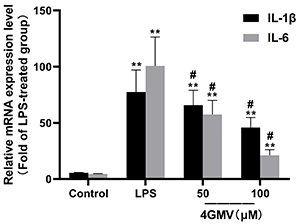
Effect of 4'-O-β-D-glucosyl-5-O-methylvisamminol (4GMV) on lipopolysaccharide (LPS)-induced mRNA expression of IL-6 and IL-1β in RAW 264.7 cells. RAW 264.7 cells were pretreated with 4GMV (50 and 100 μM) for 1 h prior to treatment with 1 μg/mL LPS. After 24 h, the expression of levels of interleukin (IL)-1β and IL-6 mRNA were measured by RT-PCR analysis. Data are reported as means±SD (n=3) of three independent experiments. **P<0.01 compared with the control group; ^#^P<0.05 compared with the LPS group (ANOVA followed by LSD).

mRNA results were consistent to cytokine assays. The results showed that treatment with LPS significantly upregulated the mRNA expressions of IL-6 and IL-1β. Compared with the LPS group, 4GMV (100 μM) had significant effect on reducing LPS-induced expression of IL-1β and IL-6 mRNA (Figure 7).

### Effect of 4GMV on NF-&mac_kgr;B and MAPK signaling pathways in LPS-stimulated RAW264.7 cells

**Figure 8:**
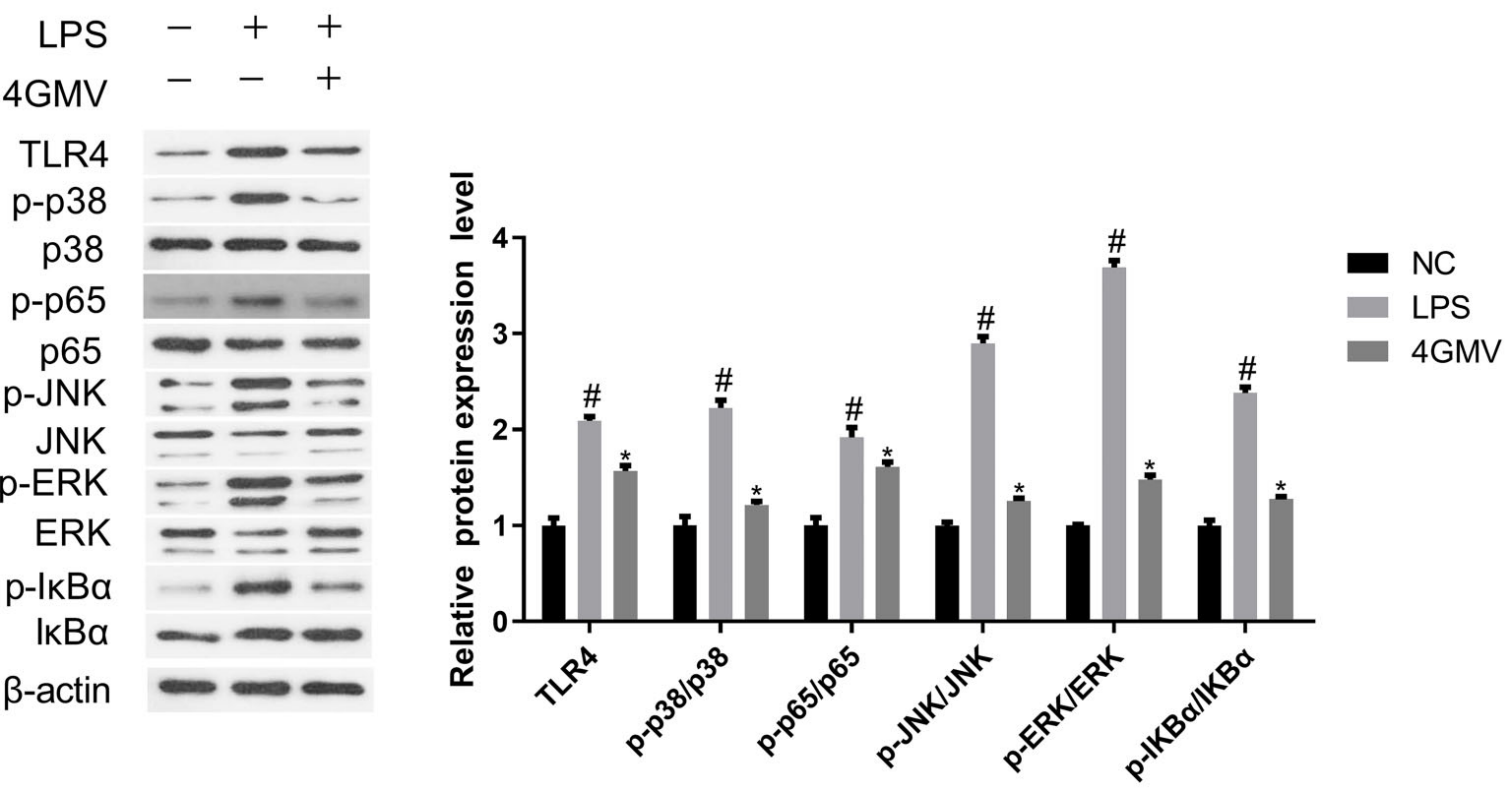
Effect of 4'-O-β-D-glucosyl-5-O-methylvisamminol (4GMV) on NF-κB and MAPK signaling pathways in lipopolysaccharide (LPS)-stimulated RAW 264.7 cells. RAW 264.7 cells were pretreated with 100 μM 4GMV and exposed to 1 μg/mL LPS for 1 h. Protein expressions of toll-like receptor 4 (TLR4), phosphorylated p38, c-Jun N-terminal kinase (JNK), extracellular signal-regulated kinase (ERK), nuclear factor of kappa light polypeptide gene enhancer in B-cells inhibitor, alpha (IκBα), and p65 were analyzed by western blot. Data are reported as means±SD (n=3) of three independent experiments. ^#^P<0.05 *vs* control; *P<0.05 *vs* the LPS-treated group (ANOVA followed by LSD). NC: negative control (β-actin). NF-κB: nuclear factor kappa-light-chain-enhancer of activated B cell; MAPK: mitogen-activated protein kinase.

TLR4 is a key factor in regulating innate immune response (22). The results indicated that 4GMV exhibited a strong inhibitory effect on the protein expression of TLR4 in RAW264.7 cells when exposed to LPS. Moreover, we detected the phosphorylation of p65, a functional subunit of NF-κB complex. Compared with the control group, the expression of phosphorylation of p65 was significantly increased in the LPS group. Pretreatment with 4GMV suppressed this excessive phosphorylation. Furthermore, phosphorylation of IκBα is essential to release NF-κB from NF-κB/IκBα complex during this process. The effect of 4GMV on LPS-induced phosphorylation of IκBα was also investigated. The results showed that 4GMV significantly inhibited phosphorylation of IκBα, indicating that 4GMV could inhibit NF-κB activation and phosphorylation of p65 by reducing phosphorylation of IκBα (Figure 8). Moreover, MAPK pathway may be taken as an intermediate stage in the regulation of NF-κB activation (23). Treatment with LPS could promote the phosphorylation of JNK, p38, and ERK in RAW264.7 cells. However, the phosphorylation levels were attenuated in 4GMV-pretreated cells compared with LPS-treated cells.

## Discussion

Psoriasis is regarded as a common inflammatory skin disease that has been characterized as chronic, common, and recurring. The mechanisms involved in the genesis of psoriasis are complex. Skin inflammation and dryness are related to impaired epidermal barrier function. The destruction of the skin barrier leads to the release of proinflammatory mediators, which intensifies inflammation (24). Repeated high-dose topical application of IMQ induces inflammatory skin lesions including erythema, scaling, and keratinocyte proliferation with acanthosis (25). Therefore, the IMQ-induced mice model has been widely applied in studies of psoriasis skin inflammation. After 7 days, typical erythema, scaling, and thickening were found in IMQ-induced skin lesions, similar to that observed in our previous study (17). After pretreatment with 4GMV, the PASI scores and the thickened epidermis were significantly attenuated in the psoriasis-like mice model. In addition, histological analyses showed that the mice treated with 4GMV exhibited ameliorated skin conditions, including smoother epidermis, less epidermal thickening, and less parakeratosis, similar to that reported in the literature. Hence, these findings indicated that 4GMV had noticeable treatment effects on IMQ-induced psoriasis-like mice model. Furthermore, the results showed that 4GMV ameliorated keratinocyte proliferation and inflammatory infiltration in IMQ-induced psoriasis-like skin lesions in BALB/c mice.

Inflammation is a hallmark of many diseases and the continuance of this process may lead to various diseases related to acute or chronic inflammation (26). LPS is an important inflammatory molecule that activates NF-κB and subsequently increases the production of pro-inflammatory cytokines such as NO, IL-1β, and IL-6, among others. (27). NO, an important cellular signaling molecule and proinflammatory mediator, plays a key role in the pathogenesis of inflammation due to overproduction in abnormal situations (28). PGE2, another important proinflammatory factor, plays an important role in acute and chronic inflammatory diseases. The production of NO and PGE2 is regulated by iNOS and COX-2, respectively. In this study, 4GMV was proven to significantly decrease the concentration of NO and PGE2 in LPS-induced RAW264.7 cells. Consistently, 4GMV also suppressed the LPS-upregulated protein expressions of COX-2 and PGE2 in a dose-dependent manner. Furthermore, the levels of the pro-inflammatory cytokines IL-1β, IL-6, IFN-γ, and IL-22 were down-regulated while the level of IL-10 was up-regulated in the 4GMV group. IL-10 is an anti-inflammatory cytokine with pleiotropic functions in immunoregulation, such as inhibition of proinflammatory cytokine expression and downregulation of NF-κB activity (29). We explored by qRT-PCR whether 4GMV suppressed the mRNA expression of pro-inflammatory cytokines. Our data showed that the mRNA levels of IL-1β and IL-6 were increased in LPS-treated RAW264.7 cells, and these results were in accordance with previous studies (30–32). It is interesting that 4GMV decreased mRNA levels of IL-1β and IL-6 in LPS-activated RAW264.7 macrophages.

Toll-like receptors (TLRs) are the major cell-surface initiators of inflammatory responses to pathogens. It has been reported that TLR-4 is the major signaling receptor in LPS-induced inflammatory responses through the NF-κB and MAPK pathways (33). Our results showed that 4GMV could suppress the protein expression of TLR-4 when exposed to LPS. To further understand the anti-inflammatory mechanism, the effect of 4GMV on the LPS-induced activation of the NF-κB and MAPK pathways was also investigated.

NF-κB, a key transcriptional factor involved in regulating the expression of proinflammatory mediators, plays a critical role in mediating inflammatory responses (34). NF-κB normally localizes in the cytoplasm by its inhibitor IκB under normal conditions. Once stimulated with LPS and proinflammatory factors, IκBα is phosphorylated and separates from NF-κB. The free NF-κB translocates into the nucleus, where the phosphorylated subunit p65 plays an important role in triggering transcription of certain genes (35). The results demonstrated that the phosphorylation of IκBα and NF-κBp65 was markedly increased in RAW264.7 cells challenged with LPS. However, 4GMV pre-treatment suppressed LPS-induced NF-κB activation by inhibiting the phosphorylation and degeneration of IκBα and thus decreased the phosphorylation of p65 through binding to TLR-4.

It has been established that activation of MAPK may be partially responsible for LPS-induced expressions of NO, IL-1β, and IL-6 in RAW264.7 macrophages (36). The data showed that the phosphorylation of JNK, p38, and ERK were markedly reversed by 4GMV in LPS-treated RAW264.7 macrophages. 2-(2-phenylethyl) chromone derivatives isolated from Chinese Agarwood showed significant inhibition of NO production in LPS-stimulated RAW264.7 cells (37). Moreover, quercetin also exhibited anti-psoriasis effects in IMQ-induced mice, and the underlying mechanism might be intimately associated with improving anti-inflammatory status and inhibiting the activation of NF-κB signaling (38), which were consistent with our research.

These findings strongly suggest that 4GMV could alleviate psoriasis in IMQ-induced psoriasis-like mice and inhibit inflammatory cytokines through suppressing the activation of TLR4-mediated NF-κB and MAPKs signaling pathways, which might represent a novel strategy for psoriasis treatment.
